# MS-YieldStackNet: multi-source data fusion for wheat yield estimation using a stacked ensemble neural network

**DOI:** 10.7717/peerj-cs.3434

**Published:** 2026-01-13

**Authors:** Waqas Ali, Zeeshan Ramzan, Muhammad Shahbaz, Qamar Ul Zaman Bhutta, Muhammad Talha, Mohammed J. AlGhamdi

**Affiliations:** 1 Department of Computer Science, University of Engineering and Technology Lahore, Lahore, Pakistan; 2 Department of Computer Science, New Campus, University of Engineering and Technology Lahore, Lahore, Pakistan; 3 Department of Computer Engineering, University of Engineering and Technology Lahore, Lahore, Pakistan; 4 Department of Computer Science (New Campus), University of Engineering and Technology Lahore, Lahore, Pakistan; 5 College of Computing, Umm Al-Qura University, Makkah, Saudi Arabia

**Keywords:** Remote sensing, Yield estimation, Food security, Artificial intelligence, Ensemble learning, Multimodal

## Abstract

Accurate crop yield prediction is vital for ensuring food security and informing agricultural policy, particularly in wheat-dependent regions like Pakistan where manual estimation methods are labor-intensive and imprecise. This study introduces a novel algorithmic framework, MS-YieldStackNet, to predict wheat yield with high spatial resolution by integrating multispectral satellite imagery, *in-situ* soil analytics, and meteorological variables. A unified feature space is constructed using Normalized Difference Vegetation Index (NDVI) and Difference Vegetation Index (DVI), soil physicochemical attributes, and temporal climate patterns, processed through a stacked ensemble neural architecture (MS-YieldStackNet) combining three parallel feed-forward neural networks (FFNNs) and a Random Forest meta-learner. The model achieved robust performance with an R-squared of 0.81, Mean Squared Error (MSE) of 6,114.30 kg/ha, root mean squared error (RMSE) of 78.19 kg/ha, mean absolute error (MAE) of 59.07 kg/ha, and mean absolute percentage error (MAPE) of 3.55%, demonstrating its potential for precise and scalable crop yield forecasting.

## Introduction

Wheat is a cornerstone of global food security, serving as a primary staple crop and a critical component of agricultural economies, particularly in regions like Pakistan where it supports millions of livelihoods. Accurate and timely wheat yield prediction is essential for informing agricultural policy, optimizing resource allocation, and mitigating food insecurity risks amidst a growing global population (Sharifani & Amini, 2023; Pang, Chang & Chen, 2022). Traditional methods, such as manual surveys and historical data analysis, are labor-intensive, costly, and often lack the precision needed for large-scale applications (Paudel et al., 2021). Recent advancements in remote sensing and machine learning have opened new avenues for improving yield forecasting by leveraging diverse data sources, including satellite imagery, climate data, and soil properties (Cai et al., 2019; Wang et al., 2020).

Remote sensing technologies, such as multispectral and hyperspectral imagery, provide valuable insights into crop health and growth patterns through vegetation indices like NDVI and DVI (Shen et al., 2022). When combined with agrometeorological parameters (*e.g*., temperature, precipitation) and soil physicochemical attributes (*e.g*., nutrient levels, pH), these data enable a comprehensive assessment of agricultural systems (Joshi et al., 2023). Machine learning approaches, including decision trees (DT), random forests (RF), and neural networks, have been increasingly applied to model these complex datasets, demonstrating improved accuracy over traditional methods (Dhiman, Bhattacharya & Roy, 2023; Muruganantham et al., 2022). However, most studies focus on single data modalities, such as remote sensing, or apply conventional machine learning models, limiting their ability to capture the multifaceted interactions influencing wheat yield at a regional level.

Despite these advancements, there remains a critical gap in integrating multispectral satellite imagery, *in-situ* soil analytics, and meteorological variables within a unified framework that leverages advanced ensemble machine learning models for regional wheat yield prediction. Existing studies often fail to combine these diverse data sources effectively or rely on single-model approaches that may not fully capture the complexity of agroecological systems, particularly in diverse regions like Faisalabad, Pakistan. This study addresses this gap by proposing MS-YieldStackNet, a novel stacked ensemble neural architecture that integrates agrometeorological, soil, and remote sensing data to deliver precise, spatially resolved wheat yield predictions. By combining vegetation indices (NDVI, DVI), soil attributes, and climate patterns, and processing them through a stacked ensemble of FFNNs and a random forest meta-learner, our approach aims to enhance prediction accuracy and support sustainable agricultural practices.

This study focuses on the Faisalabad region of Pakistan, using a 5-year dataset of yield records, agrometeorological parameters, laboratory-tested soil samples, and multispectral imagery. The dataset underwent preprocessing, which involved assessing correlations between agrometeorological parameters and yield to determine the relevance of each attribute in yield estimation. Feature selection was conducted using multi criteria approach to reduce computational complexity, mitigate the curse of dimensionality, and prevent overfitting. Pearson correlation analysis is utilised to identify statistically significant linear relationships (*p* < 0.05) between variables and yield, with a focus on parameters exhibiting $|r| > 0.3$ (*e.g*., temperature, wind speed, precipitation). Features with weak or negligible correlations $(|r| < 0.2)$ were discarded to reduce noise. Domain knowledge regarding prioritizing precipitation and temperature due to their established agroclimatic relevance was also incorporated to ensure ecologically meaningful feature retention. Subsequently, the prepared data was utilised to train five machine learning regression algorithms, including decision trees, random forest, linear regression, gradient boosting, and extreme gradient boosting. Additionally, two neural networks, namely FFNNs and convolutional neural networks (CNNs), were applied.

The research questions addressed in this study are as follows:

- **RQ1**: How can the integration of agrometeorological data, soil characteristics, remote sensing imagery, and historical yield records be leveraged to develop robust models for predicting wheat yield at the regional level?
- **RQ2**: What machine learning algorithms can be employed to analyze remote sensing data and generate accurate predictions of crop yields across large geographic regions?
- **RQ3**: How can the integration of laboratory-tested soil data enhance the precision and context-awareness of yield predictions, and what computational techniques can facilitate the incorporation of soil data into predictive models?

By addressing these questions, this work provides a scalable framework for policymakers and farmers to optimize wheat production and reinforce food security.

## Literature survey

Accurate wheat yield prediction is critical for food security and agricultural management, particularly in semi-arid regions like Pakistan. Recent studies have leveraged machine learning (ML) and remote sensing to improve forecasting, but gaps remain in integrating diverse data sources and advanced ensemble methods. This section reviews the literature under three topics: (1) ML methods for wheat yield prediction, (2) data types used in models, and (3) region-specific applications, highlighting gaps and justifying the MS-YieldStackNet approach.

### Machine learning methods for wheat yield prediction

Machine learning has transformed wheat yield prediction by modeling complex agroecological relationships. Traditional models like linear regression and decision trees have been widely used (Wang et al., 2020), but advanced methods, such as random forests and gradient boosting, offer improved accuracy, with R^2^ values up to 0.78 in semi-arid regions (Pandey & Singh, 2021). Deep learning approaches, including CNNs and long short-term memory (LSTM) models, have shown promise, achieving RMSEs as low as 522.3 kg/ha using MODIS data (Gao et al., 2022). Hybrid ML approaches, combining multiple algorithms, further enhance performance; for instance, Agarwal & Tarar (2021) integrated random forests and LSTMs for precise forecasts in Indian agriculture. Recent advancements in automated ML (AutoML) have streamlined model selection and hyperparameter tuning, with Cai et al. (2019) reporting an R^2^ of 0.75 for wheat yield using AutoML with satellite and climate data. The study Wang et al. (2020) fuse multi-source data with AdaBoost, achieving an R^2^ of 0.86 and RMSE of 0.51 t/ha for winter wheat in the U.S., utilizing high-frequency data, which is less accessible in low-resource settings. The development of an automated machine learning approach is proposed in Kheir et al. (2024) for robust and fast crop yield estimation using a fusion of soil, remote sensing, and weather dataset 20 models in the new approach, proving AutoML’s outperformance over conventional ML. The Three Decision Support System used in Kheir et al. (2025), and DSSAT wheat models slightly overestimated wheat yield but accurately predicted nitrogen content. The hybrid PBM-MLRS approach closely estimated Fe and Zn content with a root mean square error (RMSE) of 0.42 t/ha for yield and 0.89% for nitrogen content.

However, these studies often rely on single-model architectures or limited data modalities, failing to capture the full complexity of yield-influencing factors. Stacking ensemble methods, which combine multiple models (*e.g*., neural networks and random forests), remain underexplored for wheat yield prediction despite their potential to improve robustness and accuracy. This study addresses this gap by integrating three feed-forward neural networks with a random forest meta-learner, achieving an RMSE of 78.19 kg/ha and MAE of 59.07 kg/ha, surpassing prior benchmarks.

### Data types in yield prediction models

The choice of data sources significantly impacts model performance. Remote sensing data, including vegetation indices like NDVI and GNDVI, are widely used for crop monitoring, with studies reporting RMSEs of 180–850 kg/ha when combined with weather data (Pang, Chang & Chen, 2022; Ruan et al., 2022). Soil data, such as nutrient levels and pH, enhance prediction accuracy by capturing ground-level variability (Shen et al., 2022). However, most studies focus on one or two data types, limiting their ability to model complex agroecological interactions. For example, Franch et al. (2019) used remote sensing data alone, achieving an R^2^ of 0.7, while Arshad et al. (2023) improved accuracy (R^2^ = 0.85) by assimilating Sentinel-2 data into crop growth models. The lack of comprehensive multi-source integration remains a critical gap. Our study addresses this by fusing multispectral satellite imagery (NDVI, DVI), *in-situ* soil analytics, and agrometeorological data within a unified feature space, processed by MS-YieldStackNet, to achieve high precision in regional yield forecasting.

### Region-specific applications in agroecological zones

Wheat yield prediction models must account for regional agroecological variability. In semi-arid regions like Pakistan, Wang et al. (2020) developed a two-branch deep learning model for winter wheat, reporting an RMSE of 721 kg/ha. In Australia, Cai et al. (2019) fused satellite and climate data and achieved high performance with R^2^ = 0.75. In Brazil, Schwalbert et al. (2020) applied neural networks for soybean yield. These studies highlight the challenge of achieving high accuracy in diverse agroecological zones, particularly in data-scarce regions like Faisalabad, Pakistan. The proposed system outperforms these benchmarks by leveraging multi-source data and a stacked ensemble approach, achieving an R^2^ of 0.81, RMSE of 78.19 kg/ha, and MAE of 59.07 kg/ha in Faisalabad, demonstrating superior performance in a similar agroecological context.

### Novelty of this work

While prior studies have advanced wheat yield prediction, they often rely on single data modalities or conventional ML models, limiting their scalability and precision in complex agroecological settings. The proposed framework introduces a novel stacked ensemble neural architecture that integrates multispectral satellite imagery, *in-situ* soil analytics, and meteorological variables, processed through three parallel feed-forward neural networks and a random forest meta-learner. Unlike hybrid models (Agarwal & Tarar, 2021) our method optimizes multi-source data fusion and ensemble learning, achieving superior performance metrics (R^2^ = 0.81, RMSE = 78.19 kg/ha) that surpass all previous studies (see Table 1). This work advances the field by providing a scalable, high-precision framework for regional wheat yield forecasting, addressing critical gaps in data integration and model complexity.

**Table 1:** Summary of studies and proposed research on crop yield prediction.

Title	Data features	Model	Results	Study area	Crop type
Ramzan et al. (2023)	Remote sensing, weather, and yield data	Regression and neural architecture using Bayesian optimizer	MAE = 2.26 kg/ha	50 acres, Mansehra	Tea
Islam et al. (2023)	Remote sensing and weather	LR, RF, GB, and stack ensemble	MAE = 317.21 kg/ha	Terai belt, Southern Nepal	Rice
Pejak et al. (2022)	Remote sensing and soil data	MLR, SVM, RF, XGB	MAE = 4.36 kg/pixel	Upper Austria	Soya
Ayub, Khan & Haider (2022)	Remote sensing	RF and MLR	MAE = 46.14 kg/ha	Pakistan	Wheat
Ruan et al. (2022)	Remote sensing and weather	RF	RMSE = 850 kg/ha	Hebei and Jiangsu	Wheat
Shen et al. (2022)	UAV and multispectral imagery	LSTM, LSTM-RF	RMSE = 684.1 kg/ha	Henan Province	Wheat
**Proposed research**	Remote sensing, weather, soil, and yield data	RF, DT, XGB, CNN, FFNN, and stack ensemble	MAE = 59.07 kg/ha	Faisalabad, Pakistan	Wheat

## Dataset description

This section outlines the study area, data sources, and data processing procedures. The first step in the modeling process involves gathering data from three distinct sources and processing it appropriately. The proposed models are constructed using traditional regression modeling, with seven distinct regression algorithms trained and assessed using the suggested evaluation technique.

### Study area

**Figure 1:**
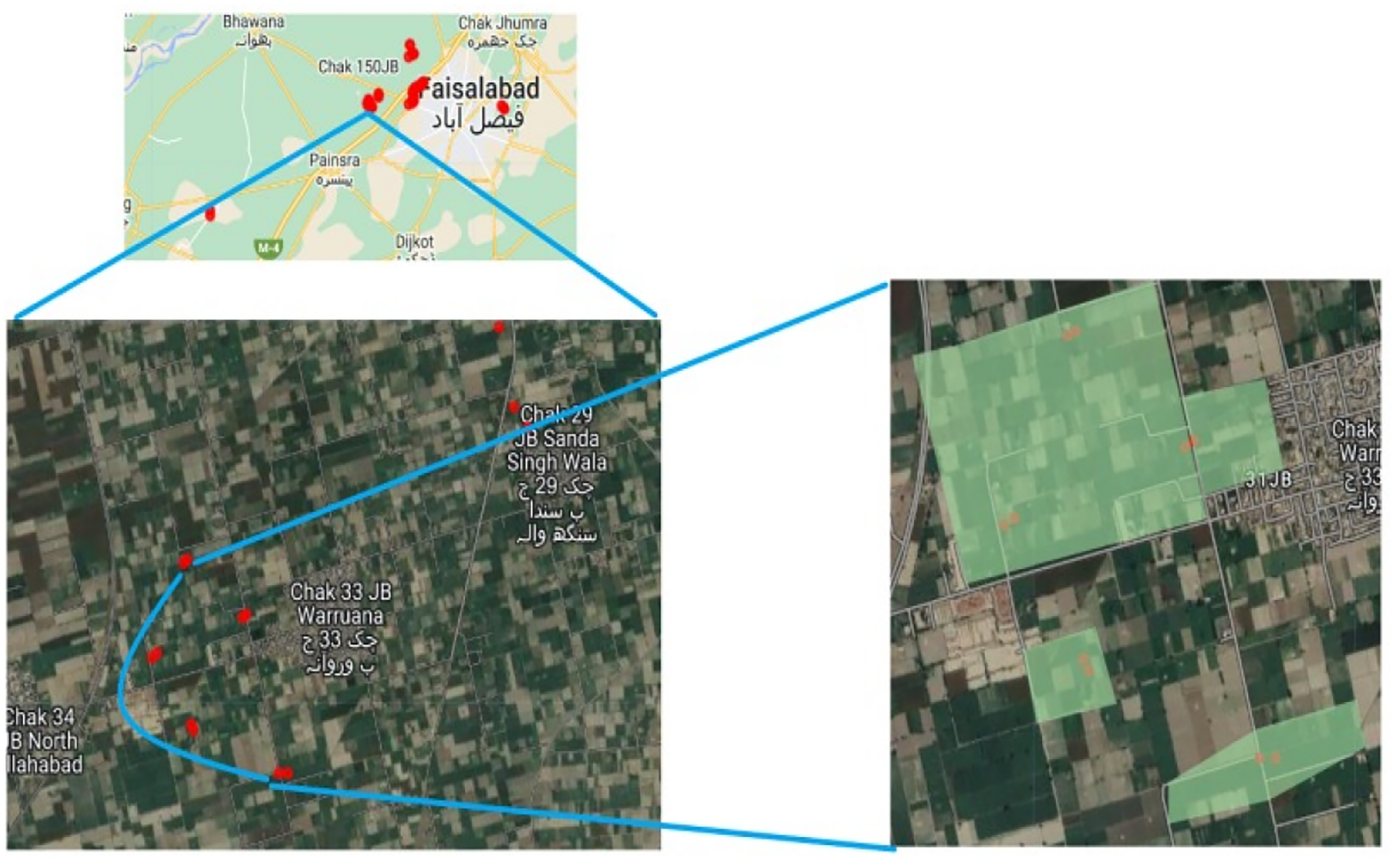
Some coordinates view using satellite imagery.

The study is carried out at approximately city level, *i.e*., Faisalabad, Pakistan. As Faisalabad is an agricultural city and a variety of crops are sown and cultivated here. The reason for selecting this area is that there are many wheat crop lands and every year these crops are cultivated. Wheat crop is considered the backbone of any country in perspective of food and economy. Also, it is considered as a cash crop as it plays a vital role in the Gross Domestic Product (GDP) of Pakistan. Agriculture University and many agriculture research centers are the plus point with respect to information gathering. The depiction of the study area using satellite imagery is represented in Fig. 1.

### Data sources

The dataset comprises wheat yield records and different agrometeorological parameters recorded seasonally for the past several years. Additionally, remote sensing data are curated to calculate vegetation indices, which, combined with agrometeorological and soil data, are utilized in constructing models for yield estimation.

#### LANDSAT-8 data modality

Landsat 8, launched by the United States (US) in February 2013, carries the Operational Land Imager (OLI) and Thermal Infrared Sensor (TIRS), recording data across various wavelengths with spatial resolutions of 15 m (panchromatic), 30 m (visible, near-infrared, shortwave infrared), and 100 m (thermal). The Landsat 8 satellite’s data is stored at the Earth Resources Observation and Science (EROS) centre, which was co-developed by National Aeronautics and Space Administration (NASA) and the U.S. Geological Survey (USGS). Multispectral data was collected *via* Landsat 7’s Enhanced Thematic Mapper Plus (ETM+) band, which is also supplied by EROS, prior to the launch of Landsat-8. The NDVI and DVI are calculated using images from precise coordinates identified *via* expert Global Positioning System’s (GPS) equipment (Google Earth Engine) throughout the growing cycle. NDVI is recognized as a superior indicator of plant health and yield potential compared to other vegetation indices.

#### Weather data

Weather data are curated from the Ayub Agriculture Research Institute, Faisalabad, and supplemented by weather websites. They have provided the weather parameters with corresponding latitude and longitude. The parameters include precipitation, minimum temperature, maximum temperature, mean temperature, relative humidity (RH), and wind-speed, recorded as seasonal averages over several years. Precipitation quantifies water (*e.g*., rain, snow) reaching the ground. Minimum and maximum temperatures represent the lowest and highest temperatures in the season, respectively, while mean temperature is the seasonal average. RH measures atmospheric moisture as a percentage, and wind-speed quantifies air movement in kilometers or miles per hour.

#### Soil data

Soil samples were collected from various fields in Faisalabad and analyzed at an agricultural laboratory in Lahore, Pakistan. Parameters extracted include organic matter, electrical conductivity (EC), potential of hydrogen (pH), available phosphorus, available potassium, saturation, and texture. EC indicates soil salinity, pH measures soil acidity or alkalinity on a scale from 0 to 14, and organic matter reflects soil fertility. Available phosphorus and potassium represent nutrients accessible to plants, while saturation and texture (proportions of sand, silt, clay) influence drainage and nutrient retention.

#### Yield data

Yield data were collected per acre from landowners and farmers at specific coordinates in Faisalabad, covering previous years. Descriptive analyses and features that have been collected during this study are detailed in Tables 2 and 3 respectively.

**Table 2:** Descriptive statistics of all features.

Feature	Count	Mean	Std	Min	25%	Median	75%	Max
Latitude	805	72.99564	0.06440	72.55	72.99321	73.00112	73.01507	73.19216
Longitude	805	31.49659	0.03659	31.273	31.4812	31.50517	31.52459	31.54178
Year	805	2020	1.41509	2018	2019	2020	2021	2022
Area	805	1	0	1	1	1	1	1
Yield	805	1,726.70	186.81	1,320	1,600	1,700	1,800	2,200
Precipitation	805	0.36562	0.27238	0.03	0.056	0.50	0.638	0.689
Min_Temp	805	9.74981	1.80622	7.6	8.1	8.6	11.2	13
Max_Temp	805	24.96298	1.85214	21	23.4	24.2	26.9	27.8
Mean_Temp	805	17.35639	1.77430	14.4	15.8	16.4	19.3	20.1
RH	805	57.14484	2.35881	53.1	55.8	56.0	60.0	61.5
Wind_Speed	805	1.63528	0.26159	1.2	1.4	1.6	1.8	2.2
DVI	804	0.158	0.059	0.047	0.110	0.155	0.202	0.340
NDVI	804	0.408	0.140	0.128	0.285	0.408	0.524	0.716
EC	805	3.024	0.578	2	2.56	3.07	3.50	4
pH	805	7.499	0.282	7	7.26	7.49	7.75	8
Organic matter	805	1.173	0.452	0.4	0.79	1.19	1.56	2
Available phosphorus	805	14.04761	3.49908	8	10.88	14.28	17.12	19.98
Available potassium	805	100.053	11.35783	80.06	89.97	100.23	109.36	120
Saturation	805	39.94118	2.90793	35.02	37.36	39.90	42.41	45

**Table 3:** Feature values.

Feature	1	2	3	4	5	6	7	8	9	10
Latitude	73.18777	73.18777	73.18777	73.18777	73.18777	73.19159	73.19159	73.19159	73.19159	73.19159
Longitude	31.44515	31.44515	31.44515	31.44515	31.44515	31.44265	31.44265	31.44265	31.44265	31.44265
Year	2018	2019	2020	2021	2022	2018	2019	2020	2021	2022
Area	1	1	1	1	1	1	1	1	1	1
Crop	WHEAT	WHEAT	WHEAT	WHEAT	WHEAT	WHEAT	WHEAT	WHEAT	WHEAT	WHEAT
Yield	1,640	1,600	1,800	1,680	2,080	1,780	1,840	1,720	1,600	1,960
Precipitation	0.04	0.4	0.056	0.638	0.64	0.03	0.6	0.057	0.614	0.64
Min_Temp	8.1	8.2	8.3	11	12.4	8	8	8.5	12	12.4
Max_Temp	23.5	23	23.6	26.4	27	22.7	22.9	24.6	26.4	27
Mean_Temp	15.8	15.6	15.95	18.7	19.7	15.35	15.45	16.55	19.2	19.7
RH	61	60.5	60.7	53.1	55.9	60.1	60.5	60.8	53.2	55.9
Wind_Speed	1.6	1.6	1.4	1.8	2	1.6	1.4	1.4	1.8	2
DVI	0.1941	0.1351	0.1451	0.1717	0.1742	0.2149	0.0935	0.1960	0.0815	0.1490
NDVI	0.4493	0.3640	0.3942	0.4294	0.4085	0.4975	0.2959	0.5267	0.2047	0.4420
EC	3.74	3.16	2.61	3.3	2.22	4	3.17	2.4	3.38	2.63
pH	7.41	7.22	7.86	7.01	7.49	7.93	7.61	7.1	7.96	7.02
Organic Matter	1.35	0.64	0.97	0.9	1.34	1.16	1.98	0.85	1.72	0.75
Available phosphorus	19.3	13.99	9.07	13.89	13.15	19.36	17.47	18.92	8.18	19.27
Available potassium	111.08	116.78	99.5	104.78	96.8	100.2	80.51	87.5	106.35	95.19
Saturation	37.08	40.98	35.73	39.29	38.4	41.96	42.36	35.43	39.34	35.17
Texture	Loam	Loam	Loam	Loam	Loam	Loam	Loam	Loam	Loam	Loam

## Data analysis and preparation

This section details the alignment, preprocessing, and analysis of data from remote sensing, weather, soil, and yield sources to construct a unified feature space for the MS-YieldStackNet model.

### Data fusion and alignment

Data from remote sensing (NDVI, DVI from Landsat-8), weather (seasonal averages of precipitation, minimum temperature, maximum temperature, mean temperature, relative humidity, wind speed), soil (organic matter, EC, pH, available phosphorus, potassium, saturation, texture), and yield (per-acre records) were aligned temporally and spatially. Temporally, data were synchronized to the wheat growing season (November–April) using seasonal aggregates over 5 years, with remote sensing images selected to match these intervals. Spatially, data were georeferenced to specific coordinates in Faisalabad using GPS equipment (aligned *via* Google Earth Engine for remote sensing data). Soil and yield data, collected at the field level, were mapped to the same coordinates. After preprocessing, features were concatenated into a single input vector per field and season, creating a unified feature space that captures agroecological interactions for the MS-YieldStackNet model.

### Handling missing data

Missing data were minimal, with only one null value each in DVI and NDVI. These rows were dropped using Python’s pandas library to maintain data integrity. No missing values were reported in weather, soil, or yield data, and the dataset contained no duplicate records.

### Correlation analysis and feature selection

**Figure 2:**
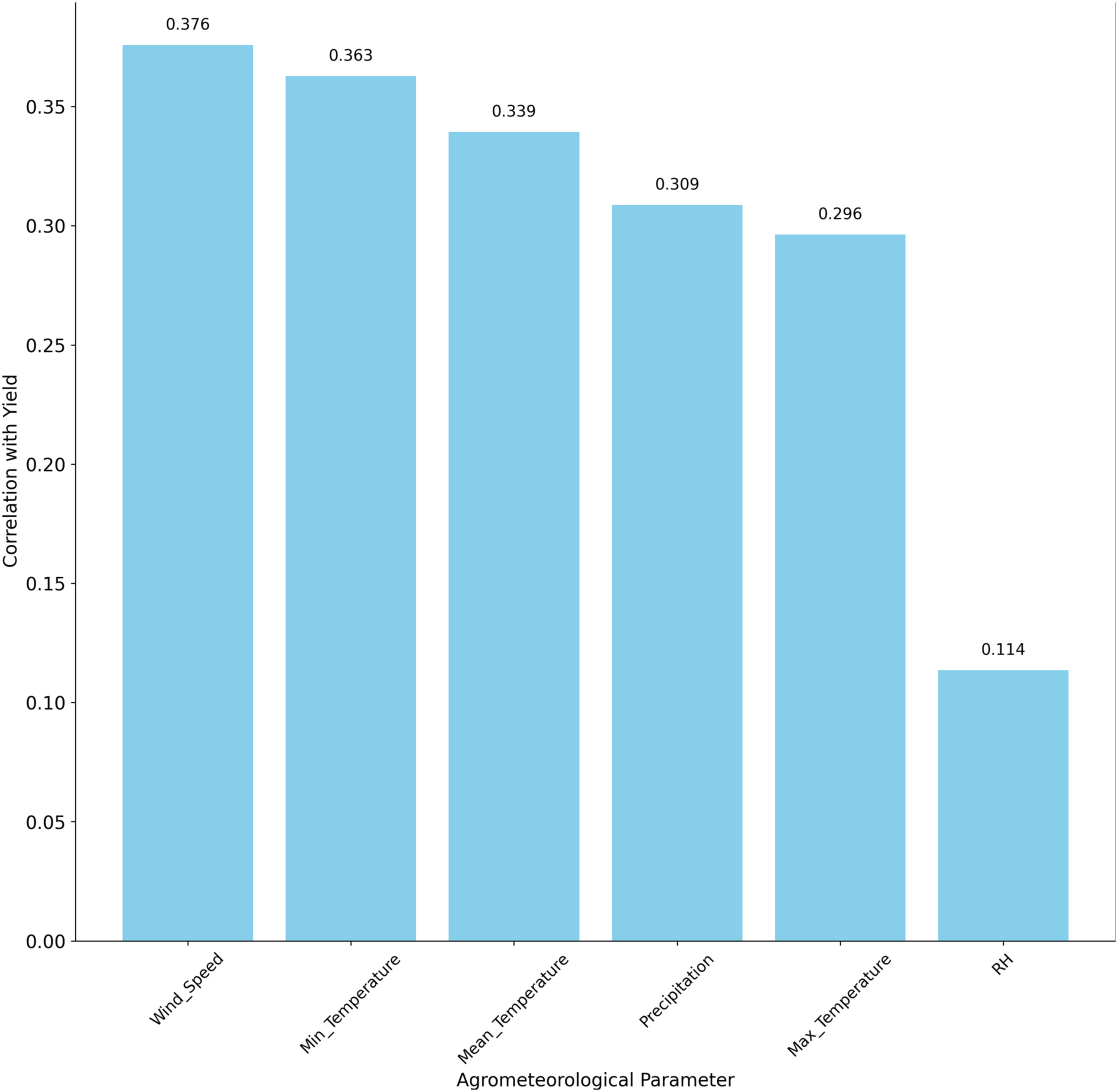
Correlating agrometeorological parameters with yield.

Subsequently, we performed a comprehensive analysis of the agrometeorological parameters that impact crop yield, as determined through Pearson correlation analysis. The correlation coefficients, ranging from −1 to 1, provide insights into the strength and direction of the relationships between each parameter and the yield. The agrometeorological parameter’s correlation with the yield is represented in Fig. 2. A threshold of $|r| > 0.3$ was selected to retain features with moderate-to-strong correlations, balancing statistical significance $(p < 0.05)$ and agroecological relevance for semi-arid regions like Faisalabad, as supported by prior studies (Pandey & Singh, 2021). For example, wind speed (r = 0.375825) and precipitation (r = 0.308777) showed positive correlations, reflecting their importance for wheat yield. Temperature attributes (maximum, minimum, mean) were also positively correlated, with minimum temperature exhibiting a strong relationship. Relative humidity also shows a positive relationship with yield, with a relatively lower correlation value, indicating that it affects the amount of wheat yield, but that the variation in humidity in the region does not significantly affect wheat growth and yield (Ismail et al., 2023). Finally, features were standardized using Z-score normalization, transforming each to have a mean of zero and a standard deviation of one, ensuring compatibility with the regression models.

## Regression modeling

**Figure 3:**
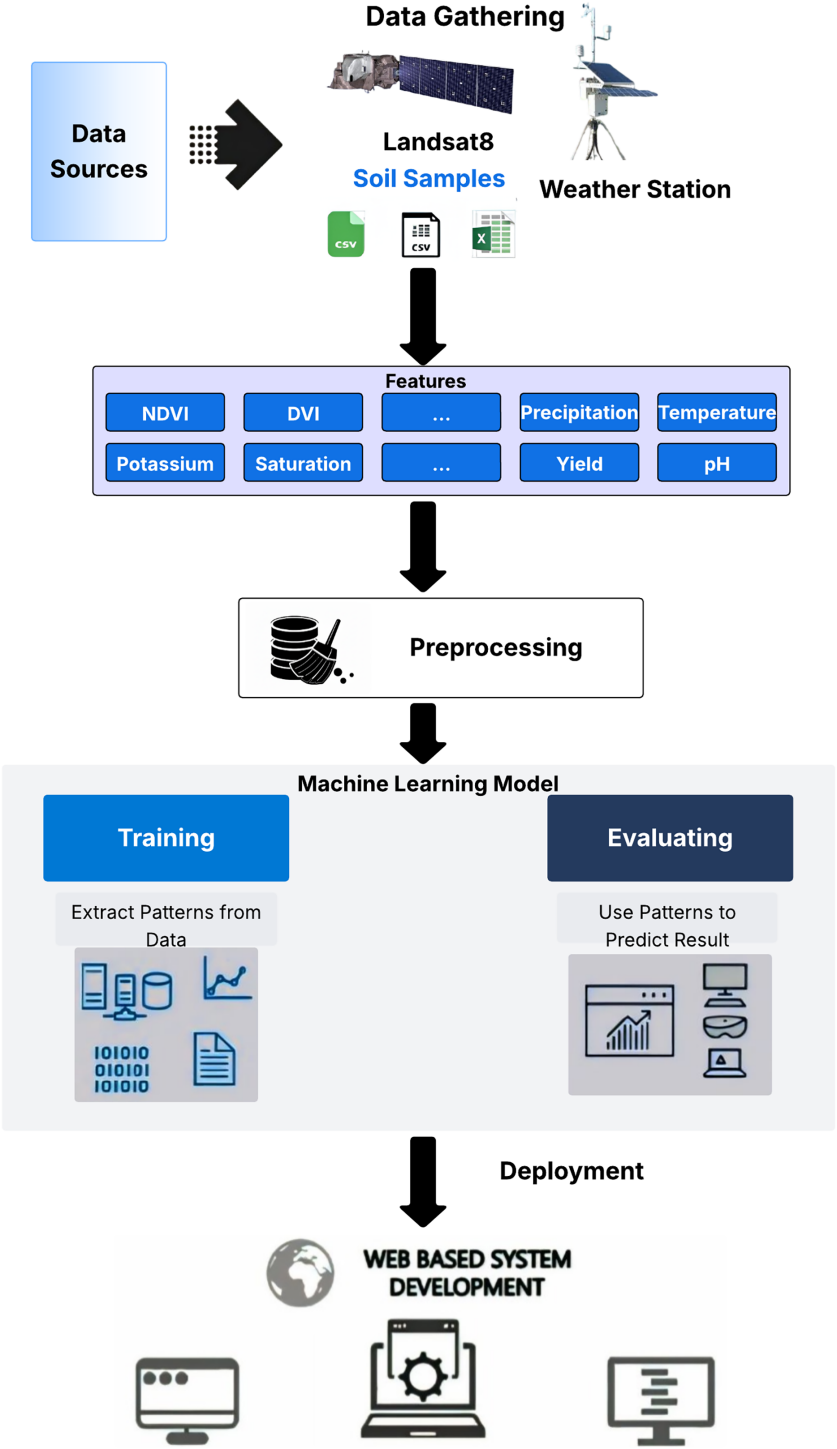
Regression modeling and evaluation framework.

Regression modeling is a robust approach for wheat yield estimation, widely utilized in state-of-the-art research (Kadam, Kanhere & Mahindrakar, 2020; Maulud & Abdulazeez, 2020). In this study, we evaluated several algorithms during the experimental phase to identify the most effective model for yield prediction. Conventional regression algorithms tested include linear regression, decision tree, random forest, gradient boosting, and extreme gradient boosting. Additionally, deep learning algorithms were assessed, including FFNNs, LSTMs, and CNNs. The proposed MS-YieldStackNet model, a stacking ensemble approach, was developed to enhance prediction accuracy. Data from multiple sources were preprocessed and used to train these models (see Fig. 3). Details of the algorithms are provided in the annexure.

### Stacking ensemble for enhanced yield prediction

The MS-YieldStackNet model addresses the research gap of limited integration of multi-source data (remote sensing, weather, soil) with advanced ensemble methods in semi-arid regions like Faisalabad, Pakistan. Its novelty lies in combining heterogeneous data with a stacking ensemble of three FFNNs and a random forest meta-learner, achieving superior performance (RMSE of 78.19 kg/ha, MAE of 59.07 kg/ha) compared to single-model approaches (Kattenborn et al., 2021; Lu, Tan & Jiang, 2021).

**Figure 4:**
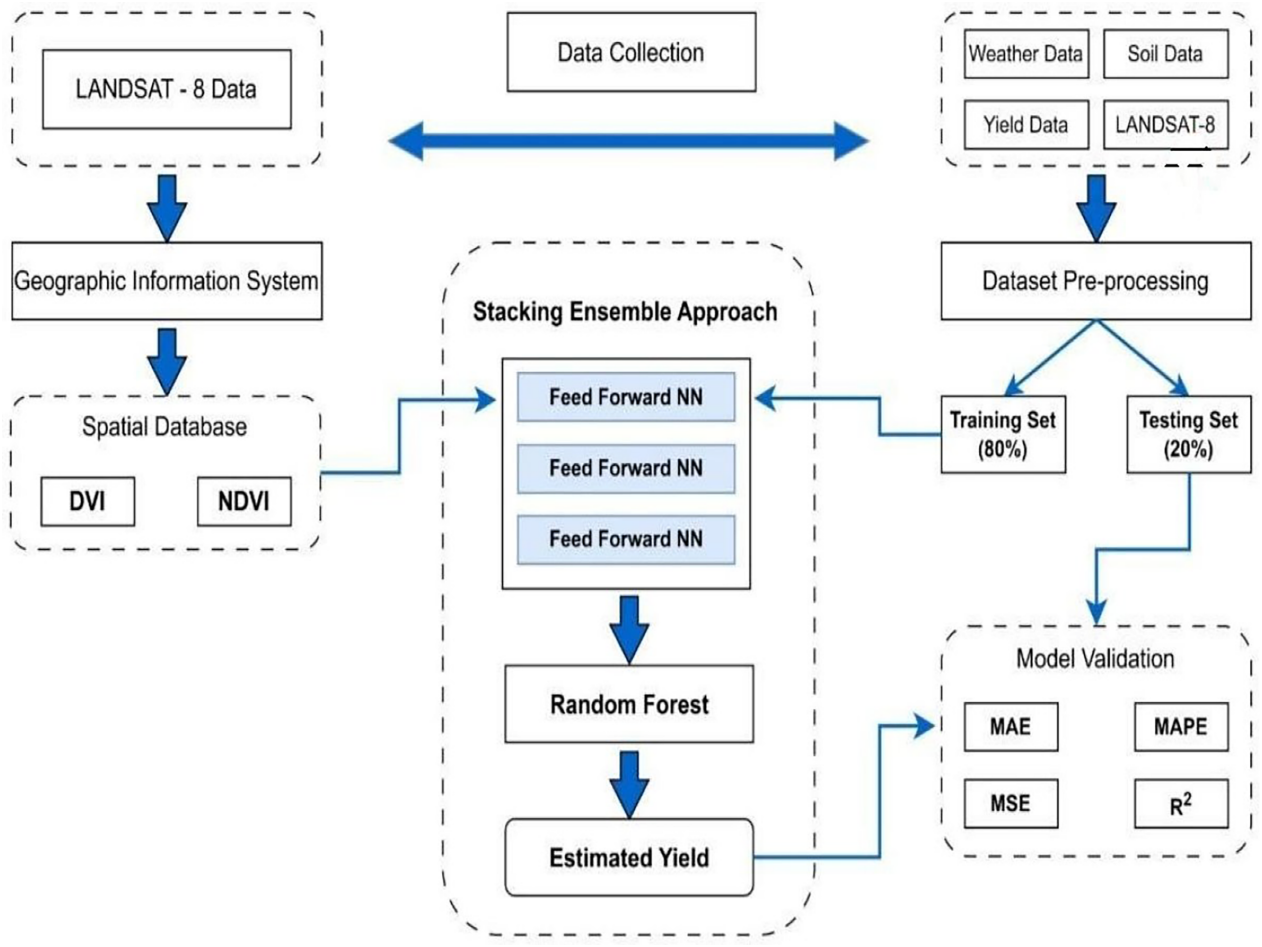
Proposed stacking ensemble architecture.

Three identical FFNNs were selected as base learners for their simplicity, computational efficiency, and ability to capture non-linear patterns in multi-source agricultural data. Empirical validation showed that three FFNNs optimized diversity and computational cost, yielding the lowest MSE (6,114.30 on the test set) compared to configurations with one, two, or four base learners. Alternative models, such as CNNs and LSTMs, underperformed due to the dataset’s limited spatial and temporal complexity, while random forest produced a higher RMSE (*e.g*., 180 kg/ha). The random forest meta-learner was chosen over a linear regressor for its robustness to overfitting and ability to model non-linear relationships among base learner predictions (see Fig. 4).

The dataset was split into 80% training and 20% testing subsets, with random shuffling applied to ensure unbiased model evaluation. Data leakage was ruled out by isolating the test set prior to training and hyperparameter tuning, ensuring it was not used to influence model development. Despite the dataset’s temporal structure (seasonal averages, November-April), random shuffling was used to align with standard machine learning practices, as the stacking ensemble effectively captured seasonal patterns.

The proposed framework for wheat yield estimation employs a comprehensive collection of multi-source data fusion and modeling strategy, formally outlined in Algorithm 1. This methodology integrates heterogeneous curated agricultural datasets comprising geospatial records ( ${{\scr D}_s}$), satellite-based vegetation indices ( ${{\scr D}_r}$), *in-situ* meteorological measurements ( ${{\scr D}_w}$), soil sample analytics ( ${{\scr D}_{soil}}$), and corresponding ground-truth yield observations ( ${{\scr D}_y}$). In the initial phase, spectral indices such as NDVI and DVI are derived from satellite imagery. Concurrently, key meteorological variables including temperature, precipitation, relative humidity, and solar radiation are collected alongside critical soil parameters (*e.g*., pH, potassium, saturation levels). These features are concatenated into a unified vector representation ${{\bf{x}}_i} \in {\mathbb  R}^{d}$ following spatio-temporal synchronization across all data modalities, resulting in a fused dataset ${{\scr D}_f}$.

**Algorithm 1:** Multi-source data fusion for wheat yield estimation using stacked ensemble neural network (MS-YieldStackNet).

** Input:** ${{\scr D}_s}$: Geospatial data from institutes (ESRI, SUPARCO, PARC);
${{\scr D}_r}$: Satellite imagery (*e.g*., Landsat-8);
${{\scr D}_w}$: Weather station data;
${{\scr D}_{soil}}$: Soil sample data;
${{\scr D}_y}$: Ground-truth yield records
** Output:** Trained model ${{\mathcal M}_{MS \; \textrm{-}YieldStackNet}}$ and predicted yield $\hat y$
1: **Step 1: Data Collection**
2: Extract vegetation indices from satellite imagery:
3: ${\rm {NDVI}} = {{NIR\;-\;RED} \over {NIR \;+\; RED}},\quad{\rm {DVI}} = NIR-RED$
4: Collect meteorological variables:
5: ${{\bf{x}}_w} = \{ T,P,RH,SR\}$
6: Collect soil properties:
7: ${{\bf{x}}_{soil}} = \{ \rm {pH} ,{\mathrm{K}} ,{\mathrm{Saturation}} , \ldots \}$
8: Combine with ground-truth yield ${{\scr D}_y}$
9: **Step 2: Feature Engineering and Fusion**
10: Construct unified feature vector:
11: ${{\bf{x}}_i} = {[{{\bf{x}}_{NDVI}},{{\bf{x}}_{DVI}},{{\bf{x}}_w},{{\bf{x}}_{soil}}]_i} \in {\mathbb R}^{d}$
12: Synchronize data sources on location and time:
13: $\textrm {Join}_{{({lat,lon,time)}}({{\cal D}_s},{{\cal D}_r},{{\cal D}_w},{{\cal D}_{soil}}) \Rightarrow {{\cal D}_f}}$
14: **Step 3: Data Preprocessing**
15: Remove noisy entries:
16: ${{\scr D}_f} \leftarrow {{\scr D}_f} \setminus \{ {x_{j}}\mid \Vert{x_{j}} - \mu \Vert > k\sigma \}$
17: Apply normalization:
18: ${{\bf{x}}_j^\prime} = {{{{\bf{x}}_j} - {\mu _{j}}} \over {{\sigma _{j}}}}$
19: Split dataset:
20: ${{\scr D}_f} = {{\scr D}_{train}} \cup {{\scr D}_{test}}$
21: **Step 4: Model Training (Stacked Ensemble)**
22: Train base learners *M*_*k*_, where $k = 1, \ldots ,K$:
23: ${M_{k}}:{{\bf{x}}_i} \to \hat y_{i}^{(k)}$
24: Concatenate outputs:
25: ${\widehat {\bf{y}}^{(base)}} = [\hat y_{i}^{(1)}, \ldots ,\hat y_{i}^{(K)}]$
26: Train meta-learner:
27: ${\hat y_{i}} = {M_{meta}}({\widehat {\bf{y}}^{(base)}})$
28: **Step 5: Evaluation**
29: Compute performance metrics:
30: ${\rm RMSE} = \sqrt {{1 \over N}\sum\limits_{i = 1}^N {{{({{\hat y}_i} - {y_{i}})}^2}} } ,\quad{R^{2}} = 1 - {{\sum {{{({y_{i}} - {{\hat y}_i})}^2}} } \over {\sum {{{({y_{i}} - \bar y)}^2}} }}$
31: **Step 6: Deployment**
32: Deploy ${{\scr M}_{SENet}}$ in a web-based platform for real-time prediction.

Subsequent preprocessing steps involve noise filtering through statistical thresholding and normalization of feature values. The dataset is then partitioned into training and testing subsets. A Stacked Ensemble Neural Network is employed for model training, where multiple base learners ${M_{k}}:{{\bf{x}}_i} \to \hat y_{i}^{(k)}$ are independently trained to generate preliminary yield predictions. Their outputs are aggregated into a meta-feature vector ${\widehat {\bf{y}}^{(base)}}$, which is then passed to a meta-learner ${M_{meta}}$ to refine the final yield estimate ${\hat y_{i}}$. Model evaluation is conducted using standard metrics, including root mean square error (RMSE) and the coefficient of determination ${R^{2}}$, ensuring predictive accuracy and generalizability. Finally, the trained model ${{\scr M}_{SENet}}$ is deployed through a web-based platform to facilitate real-time, scalable yield forecasting, thereby empowering farmers, agronomists, and policymakers with actionable insights.

### Neural architecture with hyperparameters

The structure starts with an input layer with 64 neurons, each corresponding to a feature inside the dataset. These neurons feed their activations through Rectified Linear Unit (ReLU) activation functions, which introduce non-linearity into the model. ReLU is chosen for its simplicity and effectiveness in preventing the vanishing gradient problem, commonly encountered in deep neural networks (Elsken, Metzen & Hutter, 2019; Feurer & Hutter, 2019). Following the primary hidden layer, there is a second hidden layer with 32 neurons. This layer serves to further extract and abstract features from the data, allowing the model to learn more complex patterns. Again, ReLU activation functions are applied. The output layer consists of a single neuron, configured with a linear activation function, which is suitable for continuous value regression tasks (Wistuba, Rawat & Pedapati, 2019). Following the neural network layers, a random forest model is used as a meta-learner for prediction. The choice to use Random Forest after the neural network layers is due to its robustness to overfitting, ability to handle large datasets with high dimensionality, and capability to capture complex non-linear relationships. Additionally, random forest provides interpretability through feature importance analysis, complementing the deep learning model’s predictions. The description pf model is illustrated in Table 4.

**Table 4:** Description of selected hyperparameters.

Hyperparameters	Description
Number of layers	3 (including input and output layers)
Hidden layer sizes	[64, 32]
Activation functions	ReLU for all hidden layers, Linear for output
Optimizer	Adam
Random forest	Number of trees, max depth, min samples split, *etc*.

## Results and discussion

In this section, the performance of proposed model is evaluated with evaluation metrics. Also, the performance of several predictive modeling techniques are evaluated and compared with our proposed approach.

### Evaluation metrics

Evaluation metrics provide numerical measures of a model’s performance. Evaluation metrics aid in decision-making processes such as model selection, feature engineering, hyperparameter tuning, and assessing the impact of different algorithms or techniques. While numerous metrics exist for evaluating regression models, the two most common are MSE and MAE.

MAE provides a simple understanding of the magnitude of errors in the predictions. MSE boosts large errors due to the squaring operation, making it sensitive to outliers. MAPE is particularly useful in time series analysis, where you are trying to predict future values based on historical data. By calculating the MAPE, you get a sense of how far off your forecasts are from the actual outcomes expressed as a percentage. R^2^ is useful for understanding how well the model explains the variability in the data and comparing different models’ performances. Both MSE and MAE measure the differences between predicted and actual values. However, MSE squares these differences, amplifying large errors and potentially biasing results. MAE avoids this by using absolute values, providing a more robust measure for datasets with outliers. RMSE balances emphasis on smaller and larger errors by taking the square root of MSE. Detailed descriptions and formulas for these metrics are provided in the appendix. The predictive performance of the regression algorithms is presented in Table 5.

**Table 5:** Evaluation metrics of various regression models.

Regression models	MAE	MSE	RMSE	MAPE	R^2^
LR	124.95	30,122.38	173.56	7.53	0.11
DTR	183.14	65,808.26	256.53	10.70	−0.58
RFR	120.07	29,614.68	172.09	7.20	0.13
GB	135.38	35,105.11	187.36	8.07	−0.03
XGBoost	131.58	34,168.56	184.85	7.83	0.00
FFNN	130.63	32,082.89	179.12	7.82	0.06
CNN	132.13	32,113.04	179.20	7.92	0.06
LSTM	122.76	30,155.16	173.65	7.39	0.11
Stacking ensemble	59.07	6,114.30	78.19	3.55	0.81

### Experimental results and discussion

The comparison in Table 5. demonstrates that the stacking ensemble exhibits superior predictive performance compared to baseline models, justifying its selection as the final model. The stacking ensemble achieved an R^2^ of 0.81, implying it explains nearly 81% of the observed yield variance, a strong indicator of suitability for yield prediction in this context. Performance measurements like RMSE (78.19 kg/ha), MSE (6,114.30), MAE (59.07 kg/ha), and MAPE were calculated to measure the error between predicted and actual yield values, with the stacking ensemble providing the lowest values among tested models.

**Figure 5:**
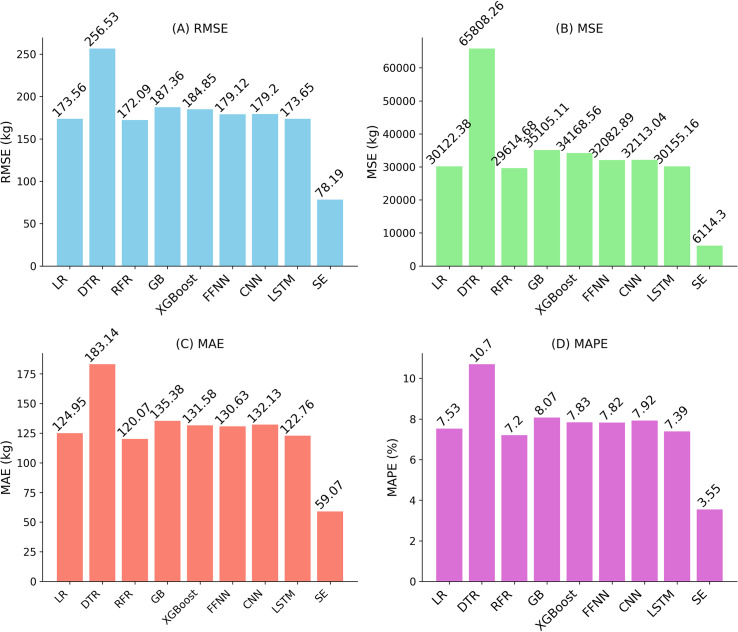
Performance evaluation of regression algorithms using (A) RMSE, (B) MSE, (C) MAE, and (D) MAPE. Lower values indicate better predictive performance.

Baseline models were included to provide a robust comparison. Linear regression, as a simple baseline, achieved RMSE of approximately 173 kg/ha, reflecting its limitations with the complex, non-linear relationships in the multi-source data. Random Forest Regression, the second-best performing algorithm, had an RMSE of approximately 172 kg/ha, while LSTM, ranked third, showed competitive performance based on MSE, RMSE, MAE, and MAPE (see Fig. 5). The comparison in Table 5 indicates that the stacking ensemble’s R^2^ of 0.81 is a reasonable value for yield prediction, reflecting its ability to capture the underlying patterns in the fused dataset.

A critical evaluation of our work against relevant and recent state-of-the-art studies confirms its performance and practical utility. For example, comparing with multi-source data and AdaBoost model by Wang et al. (2020), our method marks a reduction in prediction error. After converting their reported errors to consistent units (kg/ha) reveals that our work reduces RMSE by approximately 85% (78.19 *vs*. 510 kg/ha) and MAE by 85% (59.07 *vs*. 390 kg/ha).

Next, Kheir et al. (2024) demonstrated the effectiveness of an AutoML framework evaluating 20 models, our purpose method achieves a higher R^2^ value with a more streamlined and computationally efficient architecture Finally, MS-YieldStackNet also outperforms the complex hybrid process-based ML approach by Kheir et al. (2025), which reported an RMSE of 420 kg/ha. Our RMSE of 78.19 kg/ha represents an 81% reduction in error, demonstrating that a well-designed data-driven model can surpass the yield prediction accuracy of more intricate hybrid methodologies. Beyond raw accuracy, a key advantage of our approach is its design for practicality; unlike approaches reliant on high-frequency data or multiple model integrations, it is engineered for high performance with aggregated seasonal data, enhancing its feasibility and scalability in resource-constrained agricultural environments.

**Figure 6:**
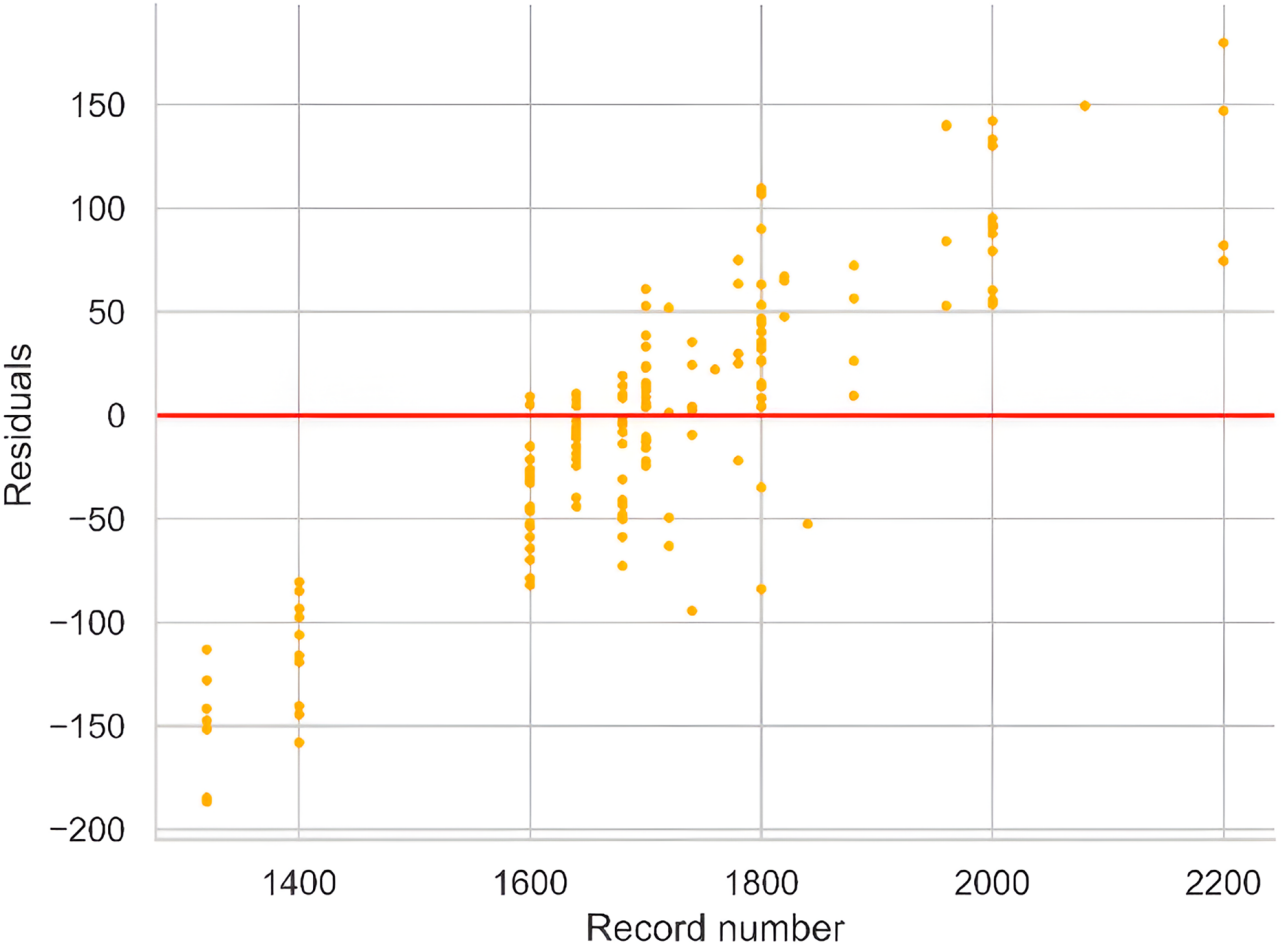
Residual plot against observations indicating the amount of error.

In an ideal scenario, the residual plot should display a random and uniform distribution of values around the identity line, serving as a critical tool for assessing the magnitude of errors and identifying observations contributing to these errors. The plot in Fig. 6 reveals heteroscedasticity, particularly for high-yield observations in the range of 2,000 to 2,200, which correspond to the most recent records. This pattern may be attributed to precision farming techniques adopted in recent years and changing agricultural methods not fully captured by the dataset attributes. These factors likely contribute to the larger residuals observed in this range, reflecting reduced correlation with historical data.

**Figure 7:**
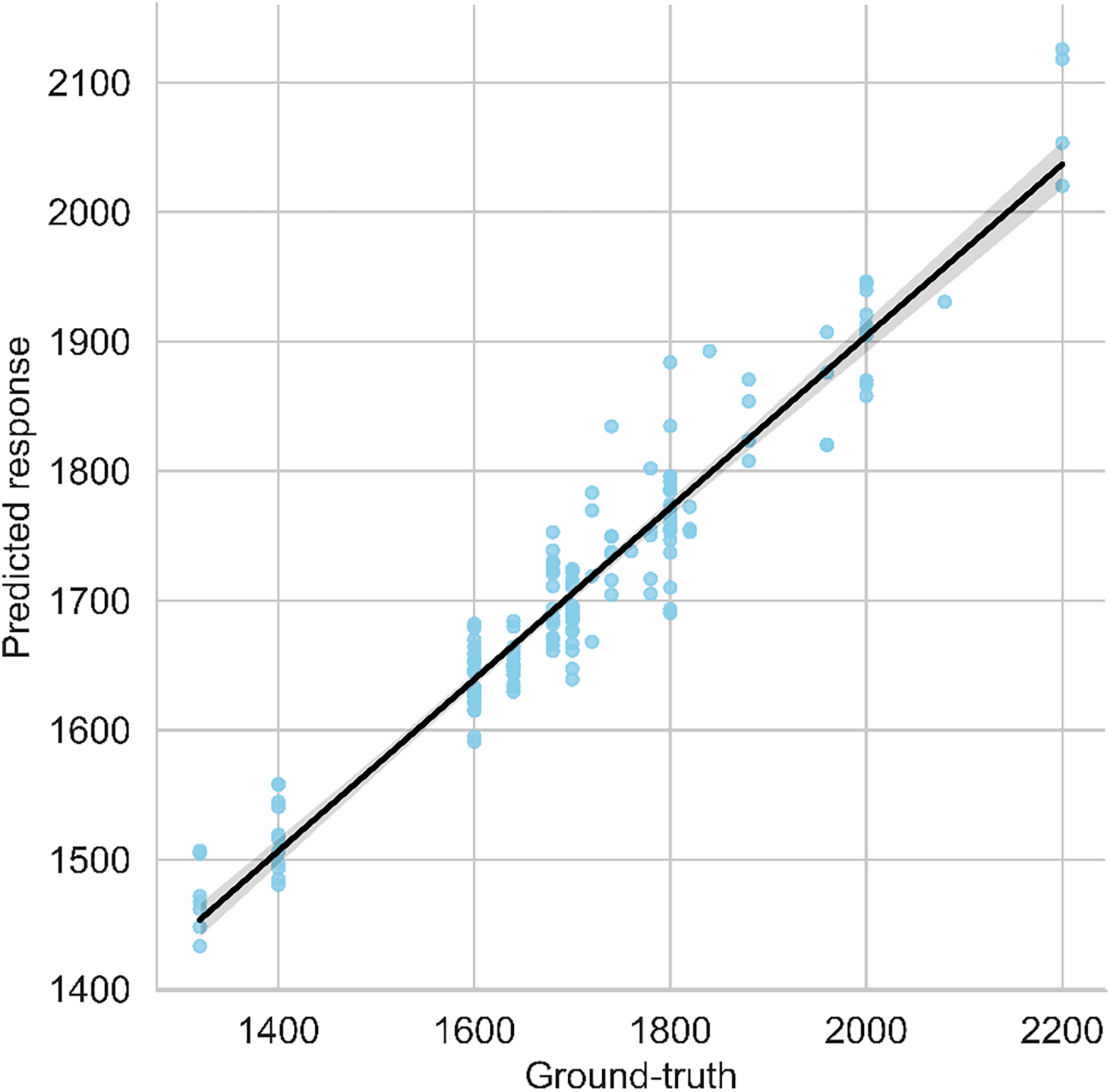
Predicted *vs*. ground-truth values plot along with best-fit regression line.

The scatter plot visualizes both anticipated and ground-reality yield values, with a regression line indicating the high-quality becoming relationship among them. A modest vertical hole among the regression line and observations suggests the version’s robustness. The graph in Fig. 7 gives a quick assessment of the model’s predictive overall performance and its alignment with real facts points. The following graph shows it carefully;

In conclusion, the stacking ensemble method showcases exceptional predictive performance, as evidenced by its high R^2^ value and low error metrics. This suggests its suitability for accurate yield prediction, outperforming other regression algorithms evaluated in the study.

This study analyzed and discussed different past studies and showed their pros and cons. Additionally, these existing studies were compared with the proposed study (see Table 6) and proved that how our system is beneficial, advanced and highly accurate than the existing ones.

**Table 6:** Comparative study of crop yield prediction approaches.

Study	Kheir et al. (2025)
Data features	Process-based models, remote sensing data
Model applied	Hybrid PBM-MLRS (Three decision support system models)
Results	RMSE = 420 kg/ha
Crop type	Wheat
Study	Kheir et al. (2024)
Data features	Remote sensing, soil, and weather data
Model applied	AutoML with 20-model super-learner ensemble
Results	R^2^ = 0.70, Willmott’s d = 0.82
Crop type	Multiple crops
Study	Islam et al. (2023)
Data features	Remote sensing and weather
Model applied	LR, RF, GB, and stack ensemble
Results	MAE = 317.21 kg/ha
Area under study	Terai belt region of southern lowland Nepal
Crop type	Rice
Study	Ayub, Khan & Haider (2022)
Data features	Remote sensing
Model applied	RF and MLR
Results	MAE = 46.14 kg/ha
Area under study	Pakistan
Crop type	Wheat
Study	Ruan et al. (2022)
Data features	Remote sensing and weather
Model applied	RF
Results	RMSE = 850 kg/ha
Area under study	Hebei and Jiangsu
Crop type	Wheat
Study	Pejak et al. (2022)
Data features	UAV and multispectral imagery
Model applied	LSTM, LSTM-RF
Results	RMSE = 684.1 kg/ha
Area under study	Henan Province
Crop type	Wheat
Study	Shen et al. (2022)
Data features	MODIS data
Model applied	LSTM
Results	RMSE = 522.3 kg/ha
Area under study	China
Crop type	Wheat
Study	Wang et al. (2020)
Data features	Multi-source satellite, weather, and soil data
Model applied	AdaBoost ensemble
Results	RMSE = 510 kg/ha, MAE = 390 kg/ha, R^2^ = 0.86
Area under study	Conterminous United States
Crop type	Winter Wheat
Study	**Proposed research**
Data features	Remote Sensing, Weather, Soil, and Yield Data
Model applied	RF, DT, XGB, CNN, FFNN, and Stack Ensemble
Results	MAE = 59.07 kg/ha
Area under study	Faisalabad, Pakistan
Crop type	Wheat

## Conclusion

This study introduces the MS-YieldStackNet model, which integrates multi-source data, remote sensing (NDVI, DVI), meteorological (seasonal averages), and soil analytics to estimate wheat yields in Faisalabad, Pakistan, achieving an R^2^ of 0.81, a MAE of 59.07 kg/ha, and a standard deviation of prediction errors of 72.99 kg/ha. The model effectively captures agroecological interactions, outperforming baseline models such as random forest regression and linear regression within this framework. As Faisalabad, a region in a developing country, represents a low-resource environment, the study demonstrates the model’s feasibility in such a setting, though training the stacking ensemble requires moderate computational resources that may challenge scalability without optimized infrastructure. The possibility of adapting MS-YieldStackNet to other regions, including Saudi Arabia, is noted, though this requires validation with region-specific data. A key limitation is the availability of data, restricted to seasonal averages over 5 years, which limits temporal resolution. Future work could focus on broader benchmarking and the use of high-resolution datasets to enhance the model’s applicability.

## Appendix

### Conventional regression algorithms

#### Decision tree

The decision tree method is one of the most broadly used and successful approaches within supervised learning. It can be used to solve problems involving both regression and classification. It is a predictor that takes the form of a tree and has three distinct classes of nodes: root node, internal nodes, and leaf nodes. The root node represents the entire sample and can be partitioned based on criteria such as entropy and information gain. Branches represent the decision rules, internal nodes represent dataset features, and leaf nodes represent the regression or classification outcomes.

#### Random forest

Random forests are one of the most popularly employed bagging ensemble approaches, used for both regression and classification strategies. They work by building multiple decision trees based on bootstrapped samples of training data. The output is obtained by averaging the values provided by the candidate models. Random forests overcome the overfitting issues common in decision trees (Hastie, Tibshirani & Friedman, 2009).

#### Gradient boosting

Gradient Boosting is one of the most popular ensemble learning algorithms for regression and classification. It organizes the construction of weak models through a gradient descent approach over an objective function, gradually improving prediction accuracy.

#### Extreme gradient boosting

Extreme Gradient Boosting, or XGBoost, is a distributed gradient-boosted decision tree system. It provides an efficient and parallel boosting process and improves processing speed over traditional gradient boosting methods. XGBoost grows trees in parallel and uses a level-wise approach for better speed and accuracy.

### Deep learning algorithms

#### Multi-layer perceptron

A multi-layer perceptron (MLP) consists of layers of interconnected nodes (neurons) organized into an input layer, hidden layers, and an output layer. It introduces non-linearity through activation functions and is widely used for classification, regression, and pattern recognition.

#### Long short-term memory

A long short-term memory network is a type of recurrent neural network (RNN) capable of learning long-term dependencies. It uses input, output, and forget gates to regulate the flow of information and preserve important sequence data over time (Sharifani & Amini, 2023).

#### Stacking

Stacking aggregates the predictions of multiple base models to produce a final prediction using a meta-model. Steps involve model selection, training base models, obtaining validation set predictions, and training a meta-model on those predictions.

#### Convolutional neural network

Convolutional neural networks process data with a grid-like topology and are used mainly for visual image analysis. Convolutional layers extract local patterns, and pooling layers reduce spatial dimensions while preserving key features. CNNs are widely used in tasks like object detection and image classification (Sharifani & Amini, 2023).

### Evaluation metrics

#### Mean squared error and root mean squared error

Mean square error (MSE) measures the average squared difference between predicted and actual values. RMSE is the square root of MSE and balances smaller and larger errors. Lower values indicate better model performance.

(1) $${\rm MSE} = {1 \over n}\sum\limits_{i = 1}^n {{{({y_{i}} - {{\bar y}_i})}^2}}$$

(2) $${\rm RMSE} = \sqrt {{{\sum\nolimits_{i = 1}^n {{{({y_{i}} - {{\bar y}_i})}^2}} } \over n}}.$$

#### Mean absolute error and mean absolute percentage error

Mean absolute error (MAE) calculates the average of the absolute differences between predicted and actual values, while MAPE expresses this error as a percentage, providing an intuitive understanding of prediction accuracy.

(3) $${\rm MAE} = {{\sum\nolimits_{i = 1}^n | {y_{i}} - {{\bar y}_i}|} \over n}$$

(4) $${\rm MAPE} = \sum\limits_{i = 1}^n {\left| {{{{y_{i}} - \bar y} \over {{y_{i}}}}} \right|}.$$

#### Coefficient of determination (R^2^)

R^2^ explains how much of the variability in a dependent variable is explained by independent variables. It is useful for comparing model performances and understanding predictive accuracy.

(5) $${\rm R}{^2} = 1 - {{\sum\nolimits_{i = 1}^n {{{({y_{i}} - \hat yi)}^2}} } \over {\sum {i = {1^n}} {{({y_{i}} - \bar y)}^2}}}.$$

## Supplemental Information

10.7717/peerj-cs.3434/supp-1 Supplemental Information 1 Source Code and Data.

10.7717/peerj-cs.3434/supp-2 Supplemental Information 2 Environmental, climatic, and soil features (such as temperature, precipitation, pH, nutrients, and texture) across various years and locations for wheat crops, used to estimate agricultural yield.

## Additional Information and Declarations

### Data Availability

The following information was supplied regarding data availability:

The data and code are available in the Supplemental Files.
